# The Depression: Online Therapy Study (D:OTS)—A Pilot Study of an Internet-Based Psychodynamic Treatment for Adolescents with Low Mood in the UK, in the Context of the COVID-19 Pandemic

**DOI:** 10.3390/ijerph182412993

**Published:** 2021-12-09

**Authors:** Nick Midgley, Brenda Guerrero-Tates, Rose Mortimer, Julian Edbrooke-Childs, Jakob Mechler, Karin Lindqvist, Susan Hajkowski, Liat Leibovich, Peter Martin, Gerhard Andersson, George Vlaescu, Peter Lilliengren, Annabel Kitson, Pamela Butler-Wheelhouse, Björn Philips

**Affiliations:** 1 The Anna Freud National Centre for Children and Families, London N1 9JH, UK; rose.mortimer@annafreud.org (R.M.); julian.childs@annafreud.org (J.E.-C.); peter.martin@ucl.ac.uk (P.M.); annabel.kitson@annafreud.org (A.K.); pamela.butler-wheelhouse@annafreud.org (P.B.-W.); 2 The Division of Psychology and Language Sciences, University College London, London WC1E 6DH, UK; brenda.tates.19@ucl.ac.uk; 3 Department of Psychology, Stockholm University, 106 91 Stockholm, Sweden; jakob.mechler@psychology.su.se (J.M.); karin.lindqvist@psychology.su.se (K.L.); bjorn.philips@psychology.su.se (B.P.); 4 Derbyshire Healthcare NHS Foundation Trust, Derby DE22 3LZ, UK; susanhajkowski@gmail.com; 5 Clinical Psychology Graduate Program, Ruppin Academic Center, Hefer Valley 4025000, Israel; liat.leibovich@annafreud.org; 6 Department of Applied Health Research, University College, London WC1E 6DH, UK; 7 Department of Behavioural Sciences and Learning, Linköping University, 581 83 Linköping, Sweden; gerhard.andersson@liu.se (G.A.); george.vlaescu@liu.se (G.V.); 8 Department of Biomedical and Clinical Sciences, Linköping University, 581 83 Linköping, Sweden; 9 Department of Clinical Neuroscience, Karolinska Institute, 171 77 Solna, Sweden; 10 Department of Health Care Sciences, Ersta Bräcke Sköndal University College, 116 28 Stockholm, Sweden; p_lilliengren@hotmail.com

**Keywords:** internet-based therapy, adolescents, depression, psychodynamic

## Abstract

**Introduction:** Face-to-face therapy is unavailable to many young people with mental health difficulties in the UK. Internet-based treatments are a low-cost, flexible, and accessible option that may be acceptable to young people. This pilot study examined the feasibility, acceptability and effectiveness of an English-language adaptation of internet-based psychodynamic treatment (iPDT) for depressed adolescents, undertaken during the COVID-19 pandemic in the UK. **Methods:** A single-group, uncontrolled design was used. A total of 23 adolescents, 16–18 years old and experiencing depression, were recruited to this study. Assessments were made at baseline and end of treatment, with additional weekly assessments of depression and anxiety symptoms. **Results:** Findings showed that it was feasible to recruit to this study during the pandemic, and to deliver the iPDT model with a good level of treatment acceptability. A statistically significant reduction in depressive symptoms and emotion dysregulation was found, with large effect size, by the end of treatment. Whilst anxiety symptoms decreased, this did not reach statistical significance. **Conclusions:** The findings suggest that this English-language adaptation of iPDT, with some further revisions, is feasible to deliver and acceptable for adolescents with depression. Preliminary data indicate that iPDT appears to be effective in reducing depressive symptoms in adolescents.

## 1. Introduction

The prevalence of mental health difficulties in children and young people in England and Wales has been increasing over the last 20 years; between 2004 and 2017, anxiety, depression, and self-harm increased, particularly among teenage girls [1]. The most recent prevalence survey conducted in July 2020 found that one in six (16%) children aged 5 to 16 years have a probable mental disorder, compared to one in nine (10.8%) in 2018 [2]. There is some evidence that the COVID-19 pandemic may have made young people’s mental health problems worse. Studies have noted a moderate increase in depressive symptoms among young people, whilst psychiatric presentations to mental health services for young people across Europe were significantly lower than expected in April 2020, suggesting a possible unmet need due to the pandemic [3]. Emerging evidence suggests that the pandemic has had a deleterious impact on young people’s mental health [4]. Young people described a range of difficulties, including reduced access to mental health support, disruption to their learning, and isolation [5].

It was in the context of the first wave of the pandemic in the UK that this project developed. Our aim was to adapt and pilot a therapist-supported internet-based programme to support adolescents with depression. Despite the prevalence of mental health difficulties amongst children and young people, many do not receive the support they need, even in usual circumstances. Those young people who do meet thresholds for help from mental health services often face long waiting lists [6], and this has been exacerbated by the COVID-19 pandemic, as services restricted face-to-face appointments due to national lockdowns and social-distancing requirements [7].

There are many barriers to young people accessing mental health support. These range from a lack of service provision, long waiting times, stigma, dependency on parents/carers to attend appointments, lack of confidence in support, and concerns about confidentiality [8,9,10]. Internet-based interventions may help to complement access to specialist mental health support, possibly addressing some of these barriers [11]. Although there are now a wide range of digital mental health programmes, here, we use ‘internet-based intervention’ to mean therapeutic interventions that are accessed online from a computer or mobile device, which include content such as text, worksheets, or videos that are worked through by the client independently, sometimes with remote therapist support. Internet-based interventions are distinct from ‘live’ tele-therapy, when therapy consists of real-time conversation between the therapist and client via an online video-conferencing platform [12].

The main advantage presented by internet-based interventions is the improved accessibility offered to service users, since treatment can be accessed remotely from any location at any time of day; this may be particularly valuable to geographically isolated populations. Additionally, internet-based therapy can address barriers related to stigma or confidentiality, given that the service user can access treatment from a device such as a mobile phone, without having to necessarily disclose difficulties to parents or carers, or sometimes anonymously. It is also a cost-effective alternative given that clinicians typically do not spend as much time as in face-to-face therapy with each client. Finally, in the context of the COVID-19 pandemic, many health services restricted face-to-face appointments to limit the risk of viral transmissions, internet-based interventions are conducted remotely and so can be delivered even in the face of any social-distancing requirements.

The majority of internet-based interventions are based on cognitive behavioural therapy (CBT), and programmes using this approach have been developed addressing various disorders and target populations [13]. Overall, the evidence from research with adult samples suggests that internet-based CBT programmes have positive outcomes when compared to waiting-list or placebo control groups, and internet-based CBT has been shown to be similarly effective to face-to-face CBT interventions [11,14]. There is a similar pattern for internet-based interventions for children and young people. Evaluations of CBT-based interventions have found similar results to those seen in adult samples, showing moderate to large effects on the reduction in symptom severity when compared to control conditions [15,16,17].

Although evaluations of internet-based therapies for children and young people have shown positive results, the lack of diversity of treatment modalities offered represents a limitation for the field, especially given the importance of providing patient choice. Furthermore, studies of face-to-face therapy suggest that different patients might be helped by different types of interventions [18]. Therefore, there is a need to develop and evaluate internet-based interventions which are built on theoretical frameworks other than CBT, where face-to-face interventions based on those frameworks have empirical evidence of effectiveness. One such alternative is psychodynamic psychotherapy. Face-to-face psychodynamic therapy has been shown to be effective for treating major depression in adults [19,20] and it has been found equally effective as CBT in the treatment of depression in adolescents [21]. Internet-based treatments for adults based on psychodynamic therapy principles have shown good results [22].

To our knowledge, there has only been one internet-based psychodynamic therapy (iPDT) intervention developed for adolescents. This programme is in Swedish and has to date only been tested in Sweden, where a recent randomised clinical trial (RCT) found that iPDT was significantly more effective than a supportive control condition in reducing symptoms of depression and anxiety and results were stable at 6 month follow up [23]. The supportive control consisted of weekly monitoring of depressive symptoms, and supportive messages sent from a therapist, consisting of basic support, empathy, and validation of emotions and experiences.

Given the value and potential of internet-based interventions in general, and the positive findings associated with the iPDT treatment developed in Sweden, there is a need to adapt and further evaluate this internet-based programme in other languages and cultures, to make it more widely accessible to young people experiencing depression. This study therefore aimed to adapt and pilot as the iPDT developed by Lindqvist and colleagues [23] in a UK setting. The adaptation of the programme included translation of the text into English, and a process of cultural adaption, informed by the views of young people with experience of mental health difficulties. The specific aims of the current study were:

Aim 1 To assess recruitment and retention rates, including at a three-month follow up.
Aim 2 To examine the acceptability of the web-based platform for the target population, including levels of engagement with different elements of the treatment over the course of the intervention.
Aim 3 To examine preliminary data on the effectiveness of the intervention in helping to reduce depressive symptoms in adolescents.

On the basis of this, and building on the findings of the Swedish studies, this study aimed to identify any possible obstacles to a full-scale evaluation of the intervention in the UK context.

## 2. Methods

### 2.1. Design

This was a pilot study based at the Anna Freud National Centre in the UK, which sought to adapt and pilot iPDT for adolescents with depression. Given the aims of this pilot study, a single-group, uncontrolled, exploratory design was used.

### 2.2. Ethical Approval

This study was conducted according to the guidelines of the Declaration of Helsinki. This study received ethical approval from University College London: Project ID Number: 19095/001, approval granted 21/4/21. Informed consent was obtained from all participants in this study.

### 2.3. Recruitment and Participants

This study was advertised through several avenues, including schools and social media advertising. Young people expressed an interest in this study via the study website.

Eligibility criteria were: aged 16 to 18 years, met criteria for Major Depressive Disorder according to assessment using the Mini International Neuropsychiatric Interview 7.0 (MINI 7.0) [24,25] and had a score of 10 or above on the Quick Inventory of Depressive Symptomatology for Adolescents (QIDS-A17-SR) [26,27]. Furthermore, participants needed to have internet access through an electronic device (computer/smartphone/tablet) and to be able to read and write English without the aid of an interpreter.

Exclusion criteria were: active suicidal ideation and/or previous suicide attempts; current participation in another psychological intervention; presence of psychotropic medication not stable for at least 3 months; use of anti-depressant medication not stable for at least 1 month; other primary diagnosis; and comorbidity with any of the following: psychotic disorder, bipolar I/II disorder, antisocial personality disorder, autism-spectrum condition, or substance use disorder. Suicidal ideation and previous suicide attempts were exclusion criteria because it was felt that it would be difficult to adequately monitor risk and safeguard these particularly vulnerable young people in the context of an internet-based programme, where we had no face-to-face contact with participants and no guaranteed way to contact a parent or carer.

Young people did not need to inform or gain consent from a parent or carer in order to participate in this study as they were aged 16 or above. However, when they signed up, all young people were asked to provide the contact number and name of a parent/carer whom the study team could call in case of an emergency situation where the team were very worried about the young person’s safety, but unable to make contact with the young person directly through telephone or email. Provision of this parent/carer contact information was required for inclusion in this study.

### 2.4. Intervention

The iPDT intervention is an internet-based programme, supported by remote contact with a therapeutic support worker. The treatment is hosted on a platform which was developed in Sweden and has been used in similar internet-based treatments [28]. The intervention includes eight modules designed to be completed over 10 weeks. The modules include videos and text on a specific topic, complemented by worksheets that young people complete and send to their therapeutic support worker, who provides feedback messages in the following days. Furthermore, the participants have a 30 min weekly text ‘chat session’ with their therapeutic support worker, using an instant-messaging platform on the therapy website.

The principal objective of the iPDT intervention is to reduce depressive symptoms through the promotion of emotional awareness and experiencing. The intervention is an affect-focused therapy and draws on Malan’s Triangle of Conflict [29], suggesting that difficult emotions trigger anxiety because they can present a threat to important relationships. The anxiety can trigger maladaptive defences against these emotions, such as avoidance, self-blame, or ‘acting out’. Young people are invited to link their emotions to depressive symptoms, to challenge defences, regulate anxiety, and explore previously avoided feelings.

The eight chapters and associated worksheets were translated from the original Swedish into English, and then went through a process of cultural adaption informed by the views of an advisory group of young people (aged 16–21) with experience of using mental health services. The young people read through sections of the chapters independently, then met as a group with members of the research team via video call to provide feedback on the length, language, and content of the chapters, as well as the images and study logo.

*Therapeutic Support Workers*: The therapeutic support workers (n = 9; 8 female, 1 male) were Masters students, all with some experience of working with children and young people, studying on academic developmental psychology programs, who received training in the therapeutic approach, involving three introductory seminars followed by a one-day practical training. Therapeutic support workers received ongoing weekly group supervision by a clinical psychologist or psychotherapist with expertise in affect-based psychodynamic therapy.

### 2.5. Measures

*Screening Measure*: The Mini-International Neuropsychiatric Interview (M.I.N.I) and the Mini-International Neuropsychiatric Interview-Kid (M.I.N.I.-Kid).

The M.I.N.I. is a structured diagnostic interview for DSM-IV and ICD-10 for people ages 18 and over; the M.I.N.I.-Kid is a version of the M.I.N.I. adapted for children and young people aged 4–17 years [24,25]. There is a specific module focussed on suicidality and suicidal risk. The interview typically takes between 15 and 60 min to complete. Interviewers were three staff members working at the Anna Freud Centre, all of whom had experience working with adolescents in a clinical or research context; two of the staff members have a research background, and the other in project management. The three interviewers received training from two clinical psychologists with experience using the M.I.N.I. and M.I.N.I.-Kid for research and clinical purposes. Interviews took place via telephone call. The interview was used to ensure that inclusion and exclusion criteria were reviewed, including identification of cases involving complex comorbidity that were not suitable or were considered too high risk for an internet-based treatment. All cases were discussed at a weekly meeting attended by two senior experienced clinicians and a senior member of the research team.

*Primary Outcome Measure*: The Quick Inventory of Depressive Symptomatology in Adolescents (QIDS-A17-SR) [26,27]. This questionnaire has 17 items that capture nine domains: sleep, appetite, mood, irritability, thoughts about death, view of the self, general interest, energy level, and restlessness/agitation. Psychometric examination of the QIDS shows acceptable reliability with α = 0.69 to 0.89 [30]. The questionnaire is self-administered, and takes approximately 5–7 min to complete. The QIDS-A17-SR score ranges from 0 to 27, with a greater score representing greater depression severity. First, a score for each of the nine domains is calculated. Domains consist of one-item (e.g., mood) or multiple items (e.g., sleep, 4 item). The domain score is taken from the highest rated item within that domain. Second, the total score is calculated as the sum of the nine domain scores.

*Secondary Outcome Measures*: The Generalised Anxiety Disorder scale (GAD-7). This is a brief self-report questionnaire which has 7 items, each scored from 0 to 3. The scores of the 7 questions are summed to give a maximum of 21, with higher scores indicating more severe anxiety [31]. The GAD-7 has demonstrated good internal consistency (α = 0.88) and convergent validity with other anxiety disorder scales [32].

The Difficulties in Emotion Regulation Scale (DERS 16). The DERS-16 is a 16 item self-report questionnaire measuring capacity for emotion regulation [33]. The psychometric evaluation of DERS-16 shows good internal consistency (α = 0.94) and convergent validity with other measures across varied demographic groups [33,34].

### 2.6. Procedure

To register interest to participate via the study website, potential participants provided basic contact information (name, age, and a contact phone number) and were invited to complete the QIDS-A17-SR online. The QIDS-A17-SR acted as a screening tool; young people who scored below 10 received an automated message saying that the treatment program might not be suitable for them, and it provided information about alternative sources of support.

Young people who scored a 10 or above on the QIDS-A17-SR received a phone call within the next 7 days from a member of the research team, who completed the M.I.N.I. or M.I.N.I-Kid, to further assess study eligibility according to the inclusion and exclusion criteria (the M.I.N.I.-Kid was used for those aged under 18). Cases were discussed at a weekly meeting led by two senior clinicians, where decisions were made about inclusion of cases in this study. Once a participant had completed both stages of the screening process, they were then invited to complete a consent form on the study website. At this stage, they completed the baseline measures. Participants then began treatment and were assigned a therapeutic support worker based on their availability for weekly chat sessions. Participants completed weekly measures as they progressed through treatment.

At the end of treatment, and at 3 month follow up, participants completed the same set of questionnaires that were administered at baseline. At the end of treatment participants were also invited to take part in a semi-structured interview, to explore their experience of the programme. (Detailed analysis of this qualitative data will be reported in a separate paper).

Participants were in treatment for this study between January 2021 and May 2021. Participants began treatment as soon as they had completed the screening phone call and signed a consent form, which meant there was a rolling start; the first participant began treatment on 21 January 2021, and the last participant to begin treatment did so on 3 March 2021, and completed on 12 May. The UK was in a full COVID-19 lockdown for January and February, and schools reopened for most students on 8th March. The ‘stay at home’ order ended on 29 March, and on 12 April, non-essential retail opened. This means that most participants completed at least some of the treatment whilst living under a COVID-19 lockdown, though some returned to school at some point during treatment.

### 2.7. Data Analysis

In order to assess recruitment and retention rates in this study, descriptive data were examined on the number of participants expressing interest, the number of young people meeting inclusion/exclusion criteria, the number completing baseline assessment, the number giving consent to participate, and the number completing the programme and completing the 3 month follow-up measures.

To examine the acceptability and usability of the treatment platform to adolescents in the target population, we analysed responses to a modified version of the Post-Study System Usability Questionnaire (PSSUQ) [35]. Items were modified to make them more relevant to the specific platform used in this study. Levels of engagement in different elements of the intervention were assessed by examining response rates of the weekly questionnaires implemented in the online platform, completion rates for all measures at baseline, end of treatment and follow up; level of attendance to the weekly chat sessions; and time taken to complete the whole intervention

To examine preliminary evidence about the effectiveness of the intervention, we estimated the change in the primary outcome (depression) and the secondary outcomes (generalised anxiety and emotion regulation) from baseline to end of treatment, and from baseline to 3 month follow up. All participants provided complete baseline data, but some data were missing at end of treatment and at follow up. For the estimation of average change, we replaced missing outcome scores by the last available measurement. This approach (also called “last observation carried forward”, LOCF) assumes that participants did not improve after the last time they provided data. This is conservative in the sense that it is likely to lead us to an under-estimate of the average symptom improvement. LOCF is now generally recognised to obtain biased estimates of treatment effects under most conditions [36]. However, in our study, there is some justification for this method, since the participants who stopped providing outcome data generally also stopped engaging in treatment (or never started to engage). We are choosing this method to avoid making the assumption that participants that dropped out of treatment subsequently improved, and thus to give our method the best chance of avoiding an overestimate of the average change. As a sensitivity analysis, we also report estimates based on participants with complete outcome data only (“complete cases analysis”). The complete cases analysis assumes that data are missing completely at random (MCAR). We also present a third alternative analysis, using a longitudinal model, which is valid under the missing at random (MAR) assumption. This is explained further below. Effect sizes were estimated using the pre-treatment standard deviation for the calculation of Cohen’s d, and bootstrapped confidence intervals with 10,000 replications.

Using baseline and end-of-treatment data only, we fit a separate mixed-effects model for each outcome, with random intercepts and slopes for participants and therapeutic support workers, using baseline and end of treatment data. We assessed random slopes and intercepts for therapeutic support workers, but found that the variance estimates for therapeutic support worker random effects were close to zero, the therapeutic support worker-level random effects did not improve the model according to the Bayesian Information Criterion, and including these terms did not affect the substantive results. Consequently, we did not include random effects for therapeutic support workers in the final model.

In the absence of therapeutic support worker-related clustering effects, the before–after comparison using a mixed-effects model with two time points is equivalent to a paired samples *t*-test of pre- and post-treatment scores. Due to the small sample size, we used the bootstrapped *t*-test [37] for the primary outcome (QIDS-A17-SR). For the two secondary outcomes (DERS-16 and GAD-7), we used a one-sample Hotelling T^2^ test to control for inflation of type-1 error rate. For all three measures, we calculated pre–post effect sizes with bootstrapped confidence interval. The bootstrapping respected the clustering of QIDS-A17-SR scores within participants (i.e., the bootstrap sampling unit was the participant), but not within therapeutic support workers, since no differences in effectiveness between therapeutic support workers were found. Our statistical test procedures are standard for before–after comparisons. Bootstrapping is a standard method of validly estimating confidence intervals in the absence of certainty about sampling distributions, such as arises in small samples such as ours [37].

Weekly ratings of QIDS-A17-SR were analysed graphically to investigate the pattern of change over the course of the intervention, and an exploratory mixed-effects longitudinal model was fitted on all weekly ratings to obtain estimates of change parameters. We did not use this model for our primary outcome analysis, because a priori we did not know the shape of change in outcome measures over time (e.g., linear, logarithmic). Since this longitudinal mixed-effects model uses all available data, including from participants who provided only partial data, it can also be used as an alternative estimate of the average symptom change. The assumption in that case is that observations are missing at random conditional on the symptom measurements that were observed.

Quantitative data analysis was conducted in R 3.6.0 [38] using the following packages: boot, dplyr, ggplot2, ICSNP, lessR, nlme, and tidyr.

## 3. Results

### 3.1. Recruitment and Retention in This Study

**Figure 1:**
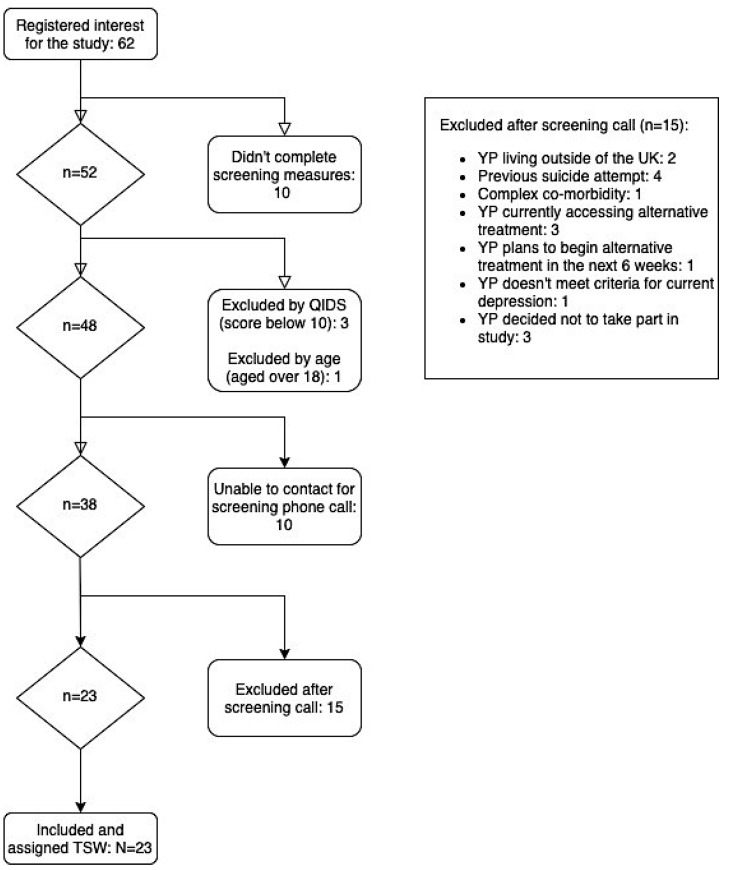
CONSORT diagram. Note. YP = young person. QIDS = Quick Inventory of Depressive Symptomatology. TSW = therapeutic support worker.

The recruitment process is detailed in the CONSORT diagram (Figure 1). Overall, 62 young people expressed an interest in this study between January and March 2021, of whom 23 (36%) were included in the program—18 female and 5 male.

Demographic information about the participants is provided in Table 1. When asked about previous use of mental health services, 12 participants (52%) had never received formal mental health treatment before. The most commonly reported barriers to receiving support were difficulties getting an appointment due to waiting lists, or not knowing where to get help.

Of the 23 participants, 2 withdrew from the programme and this study; one because they were finding the volume of reading too much in addition to schoolwork, and the other because beginning D:OTS had made them decide to seek face-to-face therapy again, as they preferred to talk rather than write. One participant withdrew after 6 days, and the other after 16 days. These participants consented to the use of the data they had provided so far.

All participants completed baseline questionnaires, and 18 out of 21 (86%) participants completed post-treatment measures upon finishing the 10 week programme. Participants completed varying numbers of their weekly measures, but more than half completed all outcome measures at all time points. Descriptive data on the three outcome measures are displayed in Table 2.

### 3.2. Acceptability of the Web-Based Platform and Levels of Engagement with Different Elements of the Treatment

At the point of joining this study, but before starting the intervention, participants were asked whether they would prefer to receive support online or face to face. A total of 14 (60%) said that it does not matter either way, 3 (13%) said that they would prefer support online, and 5 (21%) said that they would prefer face to face.

An adapted version of the PSSUQ was completed by 18 participants at the end of study, reporting levels of satisfaction with the digital platform used to deliver the intervention (the i-terapi platform). Most participants (n = 15, 83%) either agreed or strongly agreed that they were satisfied with the platform overall. Fewer participants (n = 3, 16.6%) strongly disagreed that they were satisfied with the platform overall. Generally, participants expressed most satisfaction with the ease of playing videos on the platform, the ease of correcting mistakes made in the system, and the visual design and layout of the platform. Of the three participants who expressed low satisfaction with the platform, two rated almost every aspect of the platform as very poor, responding to almost all question items with ‘strongly disagree’. Generally, participants were least satisfied with the ease of logging into the platform. Two participants provided feedback about how the platform could be improved. Both said that the one-time-passcode two-step verification was time consuming.

With regard to engagement in the treatment materials, 21/23 young people remained in this study until the end, although one did not begin the programme (either opening a chapter or having a chat session with the therapeutic support worker). All other participants engaged with at least some of the programme material. Across the 20 remaining participants, the average number of chapters opened was 7 (range: 2–8), and 13 participants opened all eight chapters. All chapters contained worksheets, which participants could fill in as they read through the chapter material. Overall engagement with the worksheets was good, although it declined over time: 19 out of 20 young people completed all of the worksheets in Chapter One, compared to 6 out of 20 for the final chapter.

Excluding the participant who had no chat sessions, the average number of chat sessions was 8.4 (range: 3–10). Whilst engagement with the chapter material and worksheets decreased over time, most participants continued to have regular chat sessions with their therapeutic support worker across the course of the programme.

### 3.3. Effectiveness of iPDT

Table 3 shows the quartiles, means, and standard deviations of self-ratings of depression, generalised anxiety, and emotion dysregulation at baseline, at the end of treatment, and at follow up. Baseline data for the two participants who withdrew before commencing treatment are included, as they would be in a per-protocol analysis of a trial. Five participants did not provide ratings at the end of treatment. At follow-up, six participants did not provide ratings on depression, and seven did not provide ratings on the secondary outcomes. These missing values were replaced by the last available measurement (LOCF, see Methods section). Complete cases information is displayed in the Supplementary, Table S1, as a sensitivity analysis.

### 3.4. Evidence of Change and Effect Size Estimation

Depression: Compared to baseline, the end-of treatment mean depression rating was lower by 4.43 points (bootstrapped 95% CI: 1.74; 7.17). The bootstrapped *t*-test yielded *p* = 0.0028 for the null hypothesis of no change. Cohen’s d was estimated as 1.12 (95% CI: 0.39; 2.01). This improvement was largely maintained at follow up, with a mean reduction compared to baseline of 3.70 points (95% CI: 1.13; 6.48, *p* = 0.007), with Cohen’s d estimated as 0.93 (95% CI: 0.26; 1.71). The results using complete cases were very similar (see Table S1).

Generalised anxiety and emotion dysregulation: The one-sample Hotelling T^2^-test of change from baseline to end-of-treatment for secondary outcome measures yielded T^2^ = 3.63, df (2, 21), *p* = 0.044. Thus, there is some evidence for change by end of treatment in at least one of the secondary outcome measures. Effect size estimates for the secondary outcomes were: GAD-7: Cohen’s d = 0.30 (95% CI: –0.49; 0.98); DERS-16: Cohen’s d = 0.84 (95% CI: 0.20; 1.53). Although follow-up means very similar to end-of-treatment means, there was slightly weaker evidence of change from baseline to follow up in secondary outcome measures (T^2^ = 2.74, df(2, 21), *p* = 0.088).

In summary, there is statistical evidence in favour of change in depression and emotion regulation by end of treatment, but not generalised anxiety by the end of treatment. There is also some evidence that treatment gains have on average been maintained at 3 months follow up.

### 3.5. Analysis of Weekly Depression Ratings

**Figure 2:**
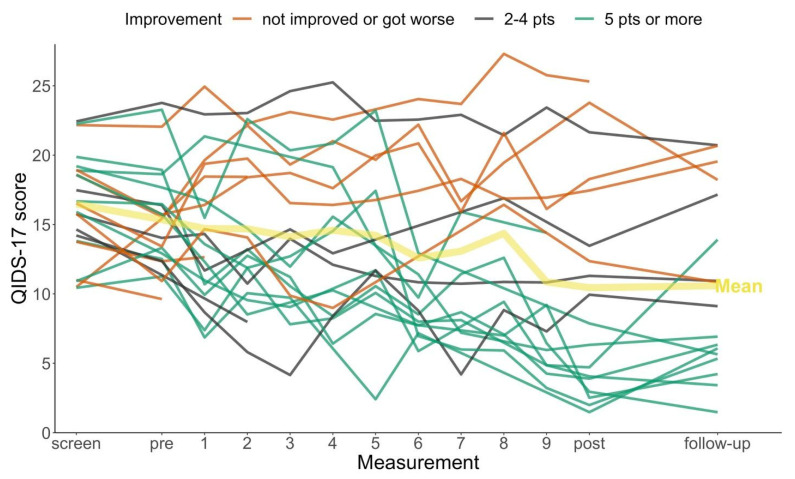
Depression self-ratings (QIDS-A17-SR) over the course of treatment (n = 23). Notes: A slight vertical random jitter was applied to make lines distinguishable in the presence of overlapping sections. Improvement is measured as the difference between QIDS-A17-SR score at “pre” minus the last available measurement. screen: Measurement at screening. pre: baseline measurement. 1–9: measurements after sessions 1–9. post: measurement after session 10 (“end of treatment”).

Figure 2 shows weekly Depression ratings of all 23 participants, along with an illustration of the mean trend over time. For visual clarity, participants were classified pragmatically into three categories, depending on how much their scores improved from baseline to end of treatment (or the last available measurement before then). Figure 2 suggests that approximately two-thirds of participants had slightly or substantially lower QIDS-A17-SR scores at the end of treatment compared to baseline (15 out of 23, or 65%, had improved by 2 points or more). The remaining one-third had the same or higher QIDS-A17-SR scores at the end (8 out of 23, or 35%). The “non-improvers” included two participants who did not provide ratings after the baseline assessment (so that they were non-improvers ‘by definition’ according to our LOCF method of replacing missing end-of treatment ratings).

There was little suggestion of non-linearity in Figure 2, so we fitted a linear longitudinal model of change from baseline to Week 10 (discarding the screening and follow-up data). We fitted two mixed-effects models: Model 1 had random intercepts and slopes for participants only, ignoring the clustering of participants within therapeutic support workers. Model 2 additionally featured random intercepts and slopes for therapeutic support workers. There was little evidence for any effect of therapeutic support workers on depression scores, since random effect estimates in Model 2 were close to zero, and Model 2 performed worse on the Bayesian Information Criterion (BIC): BIC = 982.6 for Model 1, BIC = 998.2 for Model 2. Thus, we report estimates from Model 1 in Table 4.

The results suggest a linear average improvement of QIDS-A17-SR self-ratings of between approximately 0.2 and 0.7 points per week over the 10 weeks of treatment. This model assumes that missing ratings are missing at random (MAR), which in this case would be satisfied if the missing ratings are predictable by each participant’s individual fitted regression line (via the random slope term). Under this assumption, the average treatment effect by week 10 (end of treatment) is estimated to be a reduction of 4.7 points (95% CI: 2.2; 7.3), or approximately 1.2 pre-treatment standard deviation units. This is similar to the effect sizes estimated by the LOCF method (Table 3) and the complete cases analysis (Supplementary Table S1).

**Figure 3:**
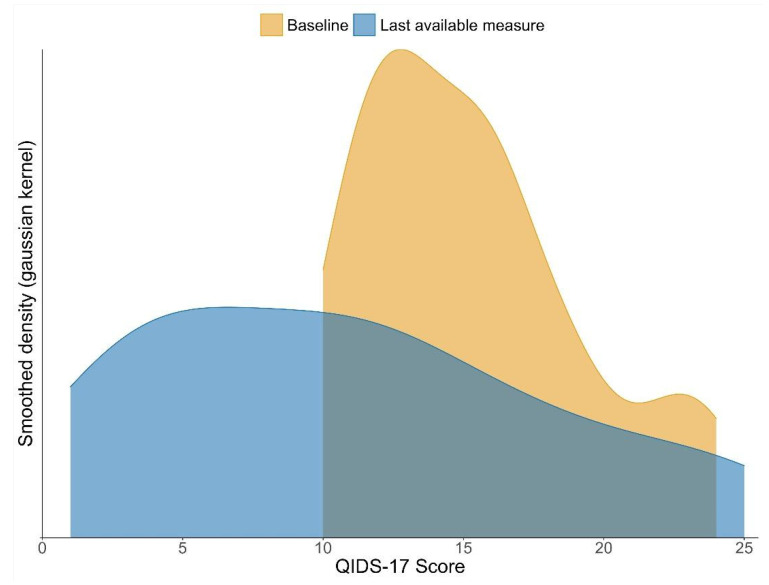
Densities of baseline and end of treatment QIDS-A17-SR ratings. Notes: This graph shows smoothed and trimmed densities estimated from the QIDS-A17-SR distributions at baseline and at the last available measurement (end of treatment or before, if end of treatment measure was not available).

To illustrate the between-participant variation around this mean improvement, Figure 3 compares the distributions of QIDS-A17-SR scores at baseline and at end of treatment (or, if that is missing, the last available measurement before then). This illustrates the shift in the distribution towards a lower mean and median, but also the observation that a substantial proportion of participants (still) had relatively high depression scores at the end of their treatment.

### 3.6. Feedback from Participants on the Impact of the Intervention

After completing the final module, young people were asked to respond to some questions about the impact of the treatment in the worksheets and had the option of writing a message to their therapeutic support worker. Nine young people completed the worksheets in the final module. There were no negative comments, and positive feedback included the following:

*I am proud of myself for completing the treatment and it’s amazing how far I have come over the weeks*

*Thank you for your guidance this has been a very important and useful journey for me to go on and I’m so grateful I got to, it’s made these past couple months much more manageable!*

*Thank you so much, you have helped me loads with recognising my thought processes and my triangle of feelings. you have been so nice to me and helped to validate my feelings so that now I can validate my own. thank you!!!*

*Thank you for everything, it has helped so much and I’m excited to carry on working on myself after this is over.*

## 4. Discussion

The aim of the present study was to adapt and pilot a therapist-supported internet-based psychodynamic programme for adolescents with depression, using an English-language version of the treatment developed by Lindqvist and colleagues [23] in Sweden. This is the first psychodynamic internet-based treatment to be developed for adolescents with depression.

The first aim of this study was to assess recruitment and retention rates. Overall, 36% of those young people who expressed an interest in this study were recruited to take part; this is comparable to the recruitment rate found in the Swedish RCT (25.9%; [23]) and reflects a reasonable ratio that could be realistically scaled up for a larger trial. Only two young people chose to leave this study, and only one was ‘disengaged’ throughout, reflecting a good retention rate, at least comparable to other internet-based interventions for youth [39].

Internet-based therapies may offer an accessible and appealing treatment option for young people, particularly those groups of young people who experience the most barriers to traditional face-to-face psychotherapy. A notable finding from this pilot is that the treatment appealed to a relatively ethnically diverse sample of young people; just over half (52%) of participants identified as White British, whilst the rest identified as Black British, a different White ethnic background, or a mixed ethnic background. In the UK, little is known about the prevalence rates of mental health disorders across various ethnic groups [40]. Whilst more children and young people who self-describe their ethnicity as ‘White British’ are diagnosed with mental health disorders than children and young people from black and minoritised ethnic groups [1], this may be because they are less likely to receive services. Indeed, research shows that young people from low socioeconomic backgrounds are more likely to experience mental health problems [1], and in turn, statistics show that minoritised ethnic groups are more likely to be classified in lower socioeconomic status that those who identify themselves as white [41]. This may suggest that black and minoritised ethnic young people experience specific barriers to seeking mental health services, relating to various intersecting types of disadvantage [42,43]. In the present study, it may be that young people from minoritised ethnic groups felt more comfortable engaging in an internet-based therapy where their own, and their therapist’s, ethnic identity is undisclosed. Young people may have felt that cultural barriers would be less pronounced in a text-based rather than face-to-face conversation, given that factors such as accent, dress, or body language are invisible and do not impact the conversation. Alternatively, they may have felt that the relationship with a therapist in internet-based therapy would be less ‘close’ and ‘personal’, and therefore that the sense of sameness and understanding provided by a shared cultural or ethnic background would not be required, since the distanced relationship required less trust than would be needed in face-to-face therapy. Finally, in the present study, young people were not required to inform parents or carers that they were participating in treatment; this may have been a facilitator for all young people, particularly those whose parents might hold negative attitudes towards the use of mental health services.

Notably, five (22%) of participants in the present study identified as male. Given that emotional disorders are more common in females than males in the 15–17 age group [1], the greater proportion of females in this study is to be expected. However, research suggests that in general, young men are less likely to access mental health support than young women [44] and young males may be more likely than females to have negative attitudes towards mental health professionals, and to view help-seeking as a sign of weakness [8]. Given this, the recruitment rates for this study are promising, and may suggest that male adolescents view internet-based therapies as a more acceptable, accessible, and less-stigmatised treatment option.

Despite these promising findings concerning the accessibility and appeal of the internet-based treatment, there are some limitations to the treatment format. To engage in treatment, young people required access to a computer or smartphone, and needed to be able to read and write in English. These requirements may make the treatment less accessible to some populations—for example, young people for whom English is not their first language, or those who do not have access to computers or mobile phones, perhaps due to poverty or other forms of disadvantage. If this treatment is found to be effective in larger trials, it would be important to develop, culturally adapt, and evaluate versions of the programme in different languages beyond Swedish and English, so that it can be accessed by a wider group of young people.

When recruiting participants to this study, we found that many of the young people who expressed an interest in taking part were presenting with higher levels of need and complexity than we had expected. A number of young people scored highly on the QIDS-A17-SR, and when completing the MINI Psychiatric Interview over the phone, reported symptoms and behaviours indicating some complexity; for example, some reported self-harming, symptoms of post-traumatic stress disorder, high levels of anxiety, or auditory hallucinations. A number of these young people told us that they were on waiting lists for Child and Adolescent Mental Health Services (CAMHS) provided by the National Health Service (NHS), but that they expected to wait several months for treatment, and saw participating in D:OTS as the only way of receiving help more quickly, or while waiting for other services to become available. When discussing such cases as a team, we found we had to balance complex clinical and ethical questions concerning whether to include young people in this study despite believing that a more intensive or multi-disciplinary treatment might be better suited to their needs, when the young people told us that alternative treatments were not immediately available. In particular, we had to consider whether we could sufficiently manage any risk issues that might occur during the course of this study—for example, if they were to harm themselves, or become distressed during chat-sessions—and balance this against the possible benefit of this study, particularly when excluding the young person could leave them with no other treatment option for a significant period of time. To manage this, in addition to reviewing and adhering to the pre-agreed safeguarding and risk-management procedures developed at the outset of this study, we implemented a weekly ‘risk-management’ meeting in which the TSWs could ‘drop-in’ and speak with a senior clinician about any concerns they had regarding the young people’s safety. Whilst some TSWs did express concern about the young people they were working with, no adverse incidents occurred, and in no cases did the study team need to use the emergency contact number provided by participants at the start of this study. Incorporating sufficient resources for weekly risk management meetings in a larger trial would be important.

Whilst some of the young people experiencing higher need and complexity did respond to treatment, some did not. This may suggest that whilst this internet-based treatment is a valuable option for some young people, for others a different type of treatment would be more appropriate. In these cases, however, barriers to treatment still exist. Whilst in the future internet-based treatments could become embedded within CAMHS services as one accessible, flexible, and low-cost treatment option, this does not eradicate the need to address other barriers to treatment, such as long waiting lists for specialist services. Such specialist services are critical for a subset of young people, and it is still very important that they can be provided to those who need them in a timely and responsive manner.

The second aim of this study was to examine the acceptability of the web-based platform for the target population, including levels of engagement with different elements of the treatment over the course of the intervention. Overall, the relatively high levels of engagement with the programme, and low numbers of people who chose to leave this study or disengage with treatment can be taken to suggest that the programme was experienced as acceptable to young people. In particular, the high levels of engagement with chat sessions suggest that young people found these to be a particularly valuable part of the programme. The percentage of participants who dropped out of the current study (13%) was similar to that reported in the Swedish clinical trial (12%), where the original version of the iPDT programme was used [23]. The evaluation of participant engagement with treatment varies across different studies, as there is not a standardised way of reporting this information, making comparison difficult. However, it appears that participant engagement in treatment in the current study is similar to that in comparable studies. The Swedish trial reported 5.8 as the mean number of modules completed out of the 8 available [23], while in the current study participants on average engaged with 7 out of 8 modules available. Other studies evaluating therapist-supported iCBT for depressed adolescents have reported similar figures concerning engagement with treatment modules, with a mean of 6.2 out of 8 modules completed [45,46]. A recent review of iCBT for anxiety and depression in young people reported that 57.2% participants who completed all the modules available [47]. Moreover, in the present study, engagement levels with the chat sessions (average of 8 out of 10; 80%) was similar to that reported in the Swedish study (6.6 out of 8; 82.5%) [23] and slightly higher than in the similar iCBT studies: 77.5% [45] and 71.3% [46]

However, there were some challenges in sustaining engagement. Whilst most participants continued to engage with chat sessions, the majority read less of the chapter material each week. During chat sessions, young people reported that they were finding it difficult to complete the chapter worksheets and read the treatment material because the end of treatment coincided with preparation for school or college exams. Given this, in the future it might be important to consider whether the treatment could be offered during school holidays, or at times when young people are unlikely to have exams or school deadlines. Additionally, it might be valuable to adapt the program to make the text more concise or turn more sections of text into audio/video, to make the program more manageable and appealing, and therefore sustain engagement across the whole ten-week treatment period.

The third aim of this study was to examine preliminary data on the effectiveness of the intervention in helping to reduce depressive symptoms in adolescents. Overall, we found statistical evidence in favour of change in depression and emotion regulation, but not generalised anxiety, by the end of treatment. There is strong evidence that the decrease in depressive symptoms was maintained at follow up, and there is evidence that the improvements in emotion regulation have also largely been maintained at follow up. These are promising findings, indicating that the internet-based intervention may be effective in the treatment of depression in adolescents, with comparable outcomes on depression compared to iCBT [45].

Although symptoms of depression and emotion dysregulation decreased over the course of the intervention, we did not find statistical evidence for a decrease in symptoms of anxiety. This was surprising, since the clinical trial conducted in Sweden found that anxiety severity had significantly decreased by the end of treatment, by an average of 4.17 points, and this was maintained at follow up [23]. A comparable study of iCBT, for adolescent depression, using a different measure of anxiety, also identified significant within-group changes but no between-group changes compared to a control condition [45]. The Swedish clinical trial of iPDT used the same measure of anxiety as the present study, so these differences between the British and the Swedish study are unlikely to be due to the measures used. It is possible that the timing of the British study may have impacted young people’s experiences of anxiety. As treatment took place during the COVID-19 pandemic, and the 10 week treatment came to an end at a time when many of the young people were preparing for school exams and returning to school following lockdown, it may be that these external circumstances caused the young people to feel heightened levels of anxiety. However, whilst anxiety symptoms did decrease slightly at follow up, this change was not statistically significant. Follow up took place during the school summer holidays, when there was no COVID-19 national lockdown in the UK; therefore, if end of treatment anxiety was a result of circumstances pertaining to school and the pandemic, we might have expected to see a significant decrease by follow up. This warrants further investigation in a randomised controlled study powered to detect treatment effects.

The findings show a greater range of QIDS-A17-SR scores by the end of treatment than compared to the beginning; whilst the mean decreased, the range increased. Descriptively, it seems that there was a group of participants for whom the treatment worked very well; these young people mostly reported very low scores at the end of treatment (a score of less than 10 on the QIDS-A17-SR), indicating that they no longer met the criteria for depression. However, there was also a group of young people—approximately one-third of all participants—for whom the treatment seemed to have very little effect, and their scores remained high over the course of the intervention. This perhaps explains the wide range of scores by the end, and points to the importance of identifying more clearly ‘what works for whom’. Future research examining mediators and moderators of outcome is called for, so as to identify which young people are likely to benefit from this treatment, and which require an alternative.

### 4.1. Strengths and Limitations

This study was the first to adapt and pilot a psychodynamic, internet-based intervention for depressed teens in a UK setting. This study was set up and delivered during the second wave of the COVID-19 pandemic in the UK, and was thus able to demonstrate the feasibility of recruiting and delivering such an intervention study, even in such adverse conditions. In using a range of reliable and validated outcome measures, which were the same as those used in the original Swedish clinical trial, direct comparison between the findings of these studies is possible. A further strength of this study was the process of cultural adaptation that took place when developing the English-language treatment, which included consultation with young people in order to review and develop both the study documentation and the treatment materials. It is hoped that this process made the treatment material more engaging and relevant to the study participants and may in part explain the high levels of engagement and retention we found. A separate paper will report on the process of cultural adaptation in more detail, in order to share learning and highlight the value of such work for other studies.

As a pilot study with no control condition or process of random allocation, there are limitations to the conclusions that can be drawn from this study. Self-selection of participants due to the self-referral process and the absence of a control group make it difficult to attribute change to the intervention itself, rather than to other confounding factors; in particular, it is difficult to know what impact the gradual reduction in COVID-19 lockdown restrictions, and associated return to school, had on participants’ depression and anxiety symptoms over the course of treatment. Further, the sample size, although sufficient for our purposes and in line with similar feasibility studies on psychological interventions [48], means that our estimates of the degree of symptom change have low precision.

Although recruitment to a randomised trial in Sweden has proved successful [23], the acceptability of randomisation in a UK context has not been demonstrated in this study, and any conclusions regarding treatment effectiveness must be made with caution. There were some indications that approximately one-third of young people were not responsive to treatment, but the sample size was too small to draw any clear conclusions regarding moderators and mediators of treatment response. These highly relevant questions must be left for a larger randomised controlled trial to address.

As a relatively complex intervention, using a mixture of text, worksheets and text-based chat sessions, it is also unclear which elements of the intervention may be most impactful on outcomes. Future studies, including dismantling trials, may help to identify, for example, how central the text-based chat sessions are to the effectiveness of iPDT. This would have important implications for practice, as the text-based chat sessions are the most resource-intensive element of the programme.

Further, the TSWs in this trial were not qualified clinical psychologists or psychotherapists, and no measure of treatment fidelity was included. Whilst the TSWs received training and weekly supervision, it is not possible to know whether treatment outcomes would have been different had the TSWs been qualified clinicians, or whether TSWs were able to deliver the iPDT model with high levels of fidelity. Inclusion of a fidelity measure in future studies would help to address this question.

### 4.2. Future Directions

In future, if the effectiveness of this internet-based treatment is established, it will be important to consider how this programme can be embedded within a wider system of mental health support for young people, including schools, colleges, and universities. One possible option would be that this treatment, and perhaps others like it, could fill the space between prevention and specialist service intervention. In order to explore this, future studies would need to examine in more detail questions concerning which young people benefit most from an internet-based treatment and which require different kinds of support.

When considering the role of internet-based treatments within the wider ecosystem of mental health support, another important consideration will be how this treatment could facilitate or support the next stage in a young person’s mental health care journey. Some young people may choose an internet-based treatment because remote contact with a therapist feels less intimidating than face-to-face therapy. For these young people, internet-based treatments may help them to feel more comfortable with psychotherapy, and therefore after completing treatment they may wish to go on to receive more support. In such a case, it might be important to consider how the internet-based treatment facilitate a smooth transition between services. For example, it might be possible for a young person to download copies of their weekly QIDS-A17-SR ratings, or transcripts of their chats with their TSW, in order to share these with a GP or mental health professional, lessening the degree to which beginning a new psychotherapy feels like ‘starting again’.

## 5. Conclusions

The findings of this pilot study suggest that an English-language version of the iPDT treatment, with some further revisions, is feasible to deliver and acceptable to adolescents with depression. Preliminary data build on the findings of a first clinical trial in Sweden, indicating that iPDT appears to be effective in reducing depressive symptoms and emotion regulation difficulties in adolescents in the UK. Further research full testing the effectiveness of the iPDT treatment is important, to provide evidence-based internet-delivered treatment options for adolescents with depression. Such innovative treatments will be one piece of the picture to provide the help adolescents need given the increasing levels of depression alongside increasing challenges in receiving services.

## Figures and Tables

**Table 1:** Participant Demographic Data.

Characteristic	N = 23
Age in years, n (%)	
16	6 (26.1)
17	7 (30.4)
18	10 (43.6)
Ethnicity, n (%)	
Black British	2 (8.69)
Different White Ethnic Background	5 (21.74)
Mixed Ethnic Background	4 (17.39)
White British	12 (52.17)
Geographical location, n (%)	
Large City	6 (26.08)
Smaller City	8 (34.78)
Countryside	9 (39.13)
Age in years of Depression Onset, n (%)	
12	3 (13%)
13	3 (13%)
14	7 (30%)
15	3 (13%)
16	7 (30%)

**Table 2:** Participant Time Statistics.

Time (Weeks)	Depression (QIDS-17)			Generalised Anxiety (GAD-7)			Emotion Dysregulation (DERS-16)		
	N	Mean	SD	N	Mean	SD	N	Mean	SD
**Screening**	23	16.48	3.70	-	-	-	-	-	-
**Baseline**	23	15.35	3.96	23	10.61	3.96	23	56.22	11.98
**1**	21	14.71	4.99	21	9.67	4.77	-	-	-
**2**	19	14.68	5.41	19	11.53	5.16	-	-	-
**3**	17	14.12	5.67	17	10.24	3.72	-	-	-
**4**	17	14.59	5.93	17	11.12	4.62	-	-	-
**5**	14	14.21	5.86	14	11.71	5.27	-	-	-
**6**	17	12.59	6.25	17	9.00	5.26	-	-	-
**7**	13	13.08	6.42	13	9.85	5.54	-	-	-
**8**	14	14.36	6.50	14	10.29	5.98	-	-	-
**9**	14	10.86	7.30	14	8.07	6.52	-	-	-
**End of treatment (10)**	18	10.44	7.85	18	8.83	6.50	18	42.56	18.58
**Follow up**	17	10.59	6.69	16	5.75	4.67	16	43.12	17.66

**Table 3:** Distribution of QIDS-17, GAD-7 and DERS-16 ratings at baseline, end of treatment, and follow up (missing values replaced by last available measurement).

	Descriptive Statistics				Cohen’s d (95% CI)		Pre–Post Test	
Outcome		Baseline	End of Treatment ^#^	Follow Up ^#^	End of Treatment	Follow Up	End of Treatment	Follow Up
Depression (QIDS-17)	1st quartile	12.5	4.5	6.0				
	Median	15.0	10.0	11.0				
	3rd quartile	17.0	15.5	17.5	1.12	0.93	t = 3.08	t = 2.62
	Mean	15.3	10.9	11.7	(0.39; 2.01)	(0.26; 1.71)	df = 22	df = 22
	SD	4.0	7.2	6.7			*p* = 0.003	*p* = 0.007
Generalised Anxiety (GAD-7)	1st quartile	8.0	3.5	2.0			T^2^ = 3.63 df(2, 21) *p* = 0.044	T^2^ = 2.74 df(2, 21) *p* = 0.088
	Median	10.0	8.0	7.0				
	3rd quartile	13.0	14.0	12.5	0.30	0.66		
	Mean	10.6	9.4	8.0	(−0.49; 0.98)	(−0.09; 1.38)		
	SD	4.0	6.5	6.2				
Emotion Regulation (DERS-16)	1st quartile	47.5	29.5	34.5				
	Median	55.0	43.0	44.0				
	3rd quartile	64.5	57.5	63.5	0.84	0.70		
	Mean	56.2	46.2	47.9	(0.22; 1.55)	(0.09; 1.40)		
	SD	12.0	18.6	18.1				
	N	23	18	17/16 *	23			23

Notes: * Follow up: N = 17 for QIDS-17, N = 16 for DERS-16 and GAD-7. ^#^ At end of treatment and follow up, missing values were substituted by the last available measurement. CI: Confidence interval (bootstrapped). Pre–post test: QIDS-17: Bootstrapped *t*-test with 10,000 samples; GAD-7 and DERS-16: Hotelling T^2^ test.

**Table 4:** Estimates from a longitudinal mixed-effects model of depression self-ratings (QIDS-A17-SR).

		Coefficient	Std Error	(95% C.I.)	
Fixed effects	Intercept	15.280	0.909		
	Time (per week)	−0.473	0.130	(−0.729;	−0.217)
		SD			
Random effects (within-participant variation)	Intercept	4.112		(2.950;	5.730)
	Slope (Time)	0.517		(0.348;	0.768)
	Correlation	0.174		(−0.349;	0.615)

Notes: n = 187, participants = 23. SD: standard deviation. C.I.: confidence interval. Time was coded 0 (baseline) to 10 (Week 10).

## Data Availability

The data presented in this study are available on request from the corresponding author. The data are not publicly available as participants did not consent to this at the outset of this study.

## Supplementary Materials

The following are available online at https://www.mdpi.com/article/10.3390/ijerph182412993/s1, Table S1: Distribution of QIDS-17, GAD-7 and DERS-16 ratings at baseline, end of treatment, and follow-up for those with complete end-of-treatment data (complete data were used for effect size estimation and statistical inference).
