# Supplementary material for: The Depression: Online Therapy Study (D:OTS)—A Pilot Study of an Internet-Based Psychodynamic Treatment for Adolescents with Low Mood in the UK, in the Context of the COVID-19 Pandemic
Source: Int J Environ Res Public Health. 2021 Dec 9;18(24):12993. doi: 10.3390/ijerph182412993 (PMC8702018; doi:10.3390/ijerph182412993)
Supplement: Supplementary file 1 [file ijerph-18-12993-s001.zip › ijerph-1448752-supplementary.pdf]

Table S1: Distribution of QIDS-17, GAD-7 and DERS-16 ratings at baseline, end of treatment, and follow-up for those with complete end-of-treatment data (complete data were used for effect size estimation and statistical inference)

		**Descriptive statistics**			**Cohen's d (95 % CI)**		**Pre-post test**	
**Outcome**		**Baseline**	**End of treatment**	**Follow-up**	**End of treatment**	**Follow-up**	**End of treatment**	**Follow-up**
Depression (QIDS-17)	1st quartile	13.0	4.0	6.6				
	Median	15.5	9.0	9.0			t = 3.07	t = 2.73
	3rd quartile	17.5	16.0	17.0	1.36	1.29	df = 17	df = 16
	Mean	15.8	10.4	10.6	(0.41; 2.48)	(0.35; 2.30)	p = 0.011	p = 0.016
	SD	4.0	7.9	6.7				
Generalized Anxiety (GAD-7)	1st quartile	8.0	3.3	2.0				
	Median	10.5	7.0	4.5				
	3rd quartile	12.8	13.8	8.0	0.42	1.38		
	Mean	10.4	8.8	5.8	(−0.63; 1.33)	(0.61; 2.25)		T^2 = 10.67
	SD	3.7	6.5	4.7			T^2 = 8.47	df(2, 14)
Emotion Regulation (DERS-16)	1st quartile	47.3	28.3	30.5			df(2, 16)	p = 0.002
	Median	54.0	37.5	39.5			p = 0.015	
	3rd quartile	62.5	47.8	59.8	1.07	1.02		
	Mean	55.4	42.6	43.1	(0.20; 1.88)	(0.13; 1.93)		
	SD	12.0	18.6	17.7				
	N	18	18	17/16 *	18	17/16*	18	17/16 *

Notes:
*Follow-up: N = 17 for QIDS-17, N = 16 for DERS-16 and GAD-7.
CI: Confidence interval (bootstrapped)
Pre-post test: QIDS-17: Bootstrapped t-test with 10,000 samples; GAD-7 and DERS-16: Hotelling T2 test.
